# Deep Neural Network Model for Approximating Eigenmodes Localized by a Confining Potential

**DOI:** 10.3390/e23010095

**Published:** 2021-01-11

**Authors:** Luka Grubišić, Marko Hajba, Domagoj Lacmanović

**Affiliations:** 1 Department of Mathematics, Faculty of Science, University of Zagreb, 10000 Zagreb, Croatia; domagoj.lacmanovic@math.hr; 2 Department of ICT, Virovitica College, 33000 Virovitica, Croatia; marko.hajba@vsmti.hr

**Keywords:** Anderson localization, deep neural networks, residual error estimates, physics informed neural networks

## Abstract

We study eigenmode localization for a class of elliptic reaction-diffusion operators. As the prototype model problem we use a family of Schrödinger Hamiltonians parametrized by random potentials and study the associated effective confining potential. This problem is posed in the finite domain and we compute localized bounded states at the lower end of the spectrum. We present several deep network architectures that predict the localization of bounded states from a sample of a potential. For tackling higher dimensional problems, we consider a class of physics-informed deep dense networks. In particular, we focus on the interpretability of the proposed approaches. Deep network is used as a general reduced order model that describes the nonlinear connection between the potential and the ground state. The performance of the surrogate reduced model is controlled by an error estimator and the model is updated if necessary. Finally, we present a host of experiments to measure the accuracy and performance of the proposed algorithm.

## 1. Introduction

In this paper we study features of the spectral problem for the family of elliptic operators of the reaction-diffusion type posed in the finite domain $\Omega ={\left[-B,B\right]}^{n}$, B>0. These operators are also known as Schrödinger operators or Schrödinger Hamiltonians and they are defined by the differential expression of the form:

$$H_{\omega }u=-\Delta u+V_{\omega }u\;.$$

Here −Δ is the distributional realisation of the Laplace operator and $V_{\omega }$ is the multiplication operator with the real function $V_{\omega }$. The parameter ω describes a random perturbation of a given potential. The associated spectral problem is to find an eigenvalue ε and an eigenmode u≠0 such that *u* verifies:

(1) $$H_{\omega }u=\varepsilon u$$

and a set of boundary conditions. We will consider boundary conditions that lead to the realization of the expression *H* as a self-adjoint operator in a Hilbert space. In particular, we will consider functions $u\in H_{0}^{1}\left(\Omega \right)$ with Dirichlet boundary conditions and $u\in H_{\pi }^{1}\left(\Omega \right)$, where $H_{\pi }^{1}\left(\Omega \right)$ is the first-order Sobolev space of functions (square integrable functions whose gradient is also square integrable) that satisfy the periodic boundary conditions [1,2]. In what follows we will use ∥·∥ to denote the $L^{2}\left(\Omega \right)$ norm of a square integrable function.

We will consider—as an academic prototype—short-range confining electrostatic potentials such as those considered in [3] (see also [1,2]) and a more challenging class of confining potentials related to the effect of Anderson localization [4]. By the effect of localization we mean that we search for eigenvalues ε such that *u*, ∥u∥=1, is essentially zero in the large part of the domain Ω. The Anderson model describes quantum states of an electron in a disordered alloy that can transition from metallic to insulating behavior. Loosely stated, we aim to model the connection $E:V_{\omega }\mapsto u_{\omega }$, where $u_{\omega }$, $u_{\omega }>0$ is the eigenmode of the lowermost ε in (1). Such eigenmodes are called the ground states, and $\varepsilon_{0}=\varepsilon$ is called the ground state energy. Note that for the elliptic reaction-diffusion operators $H_{\omega }$ defined in $L^{2}\left(\Omega \right)$ and with the potential $V_{\omega }$, which is bounded, nonnegative and positive on a set of positive measures, there exists—by an application of the Krein–Rutman Theorem—the unique ground state $u_{0}>0$, which verifies (1) and so the mapping E is well defined; see [5,6].

We emphasize that we use the following regularization approach from [6] to deal with rough potentials. Namely for $V_{\omega }$, which is bounded, nonnegative and positive on a set of positive measures there exists $u_{\omega }\in C^{1}\left(\Omega \right)$, $u_{\omega }>0$ such that $H_{\omega }u_{\omega }=1$. It can be shown by direct calculation that the operator $A_{\omega }$ defined in $H_{\pi }^{1}\left(\Omega \right)$ by the differential expression:

$$A_{\omega }\phi =-\frac{1}{u_{\omega }^{2}}\mathrm{div}\left(u_{\omega }^{2}\mathrm{grad}\phi \right)+\frac{1}{u_{\omega }}\phi$$

has the same eigenvalues as the operator $H_{\omega }$. Furthermore, ψ is an eigenmode of $H_{\omega }$ if and only if $\phi =\psi /u_{\omega }$ is an eigenmode of $A_{\omega }$. Based on this equivalence, we call the function $W=1/u_{\omega }$ the effective confining potential defined by $V_{\omega }$. For more details see [5,7].

One possibility of obtaining data-sparse representations of the solutions of elliptic problems is through the use of tensor networks also known as tensor train decompositions or matrix product states [8,9]. We choose a more direct approach known as Variational Physical Informed Neural Network (VPINN) [10,11,12]. Realizations of these dense network architectures are trained to solve the variational formulation of the problem. This approach to training neural networks is a part of the unsupervised learning paradigm. It is a mesh-less approach that is capable of solving variational (physical) problems, by minimizing the loss functional, which combines the variational energy of the system together with penalty terms implementing further physical or normalization constraints.

The main physical constraint for the ground state is the positivity constraint. To deal with excited states we need to implement further symmetry constraints in the variational space. We opt for a different approach, also based on positivity constraints and a-posteriori error estimation. We use the neural network to approximate the solution of the source problem:

−Δu+Vu=1

with associated boundary conditions. The solution *u* is called the landscape function, and in this case we are interested in the mapping L:V↦u. The landscape function is a positive function in $C^{1}\left(\Omega \right)$ and its reciprocal W=1/u is called the effective potential. The effective potential provides a mechanism that incurs localization on bounded states. To localize the excited states we use the approach of [5,7]. Let $W_{\min,i}$ be the *i*-th lowermost minimum of the effective potential *W*. It was observed in [5] that the following heuristic formula:

(2) $${\tilde{\epsilon }}_{i-1}=\left(1+\frac{n}{4}\right)W_{\min,i}$$

yields good approximations to the energies of excited states. Note that this relationship, given its simplicity, is also something we might reasonably hope to learn algorithmically from a sample of landscape functions. This was stated as a motivation to utilize neural networks in the study of the eigenvalue problem for Schrödinger operators [3].

For an eigenmode ψ of *H* with the eigenvalue ε we have the estimate: $\psi \left(x\right)\leq {\varepsilon u∥\psi ∥}_{L^{\infty }\left(\Omega \right)}$, x∈Ω. This estimate can be obtained (see [13]) using the Feynman–Kac formula for the representation of the bounded state as an expectation of an integral of the Brownian motion. It was further argued in [13] that an eigenmode ψ with energy ε:=ε(ψ) can only localize in the region:

(3) {x:εu(x)≥1}.

Subsequently, as a combination of (2) and (3) we get both information on the excited state energy and information on the location of the excited state’s support.

### The Motivation and the Contribution of this Paper

The use of neural networks as data-sparse representations of complex, high dimensional nonlinear mappings is an emerging trend in many disciplines. In particular, it has been used to tackle many body Schrödinger equations [14,15], the Black–Scholes equation, the Hamilton–Jacobi–Bellman equation, and the Allen–Cahn equation [10,11,16,17,18,19,20].

In general, all of the above problems can be reduced to computing an approximation of the function $u:\Omega \subset R^{n}\to R$. This approximation is constructed by optimizing (training) the parameters of the family of test functions (we chose the family of all realizations of a given neural network architecture) so that the value of the appropriate energy functional (for the chosen model) is minimal. The main challenge in such an approach is to assess the approximation accuracy and to ensure that the computed realization of the neural network satisfies further physical constraints, such as symmetry constraint or the boundary conditions.

Further physical, but also numerical, constraints can be built into the optimization model in several ways. The most scalable and flexible way is to use penalization [10,11,20,21]. A alternative more subtle, and more accurate way is to introduce the constraints directly into the family of test functions as it is done in the architecture of the PauliNet from [14] (see also [22,23]), or to construct a family of test functions using an ansatz that combines several components of the solution, which are themselves realizations of neural networks [12,24].

In this study we focus on the potentials for which the Hamiltonian satisfies the Krein–Rutman theorem (the scaled ground state is the unique positive and smooth function). Examples of such potentials are the effective potentials associated with the Anderson localization. Since this is a more restrictive class of potentials than those considered in [14], we opt for a direct approach. Our contribution is the introduction of the residual error estimator into the Deep Ritz Algorithm from [20]. This in turn allows us to use Temple–Kato [25,26] or similar inequalities [27,28] to ascertain if the ground state generated by the neural network is a certified small perturbation of a physical eigenstate. For activation functions that are smooth enough we can calculate the strong form of the eigenvalue residual and then compute its norm using a quadrature or quasi-Monte Carlo integration. Using the residual estimator we stop the optimization (training process) when the eigenvalue residual is small enough (satisfies the preset tolerance) and/or the convergence criterion for the optimization algorithm is met (Adam optimizer). The use of ansatz functions based on neural networks, such as [24], will undoubtedly be a method of choice for 2D or 3D problems. However, this method depends on an accurate representation of the boundary of the domain Ω and thus faces challenges in scaling to higher dimensions.

The treatment of physical symmetries becomes critical when approximating excited states. For dealing with this task, we reformulate the problem as an inverse problem based on the solution of the source problem Hu=1. The main constraint that the solution must satisfy is again positivity, and we construct an error estimator to certify the quality of the solution.

The network architectures used so far are dense network architectures. Inspired by [3], we study a parameter-dependent family of potentials and present a fully convolutional encoder–decoder neural network as a reduced order (surrogate) model for this family of partial differential equations and the mapping L:V↦u. We formulate a new certified surrogate modeling approach based on neural networks that is inspired by the work on certified surrogate modeling from [29] and the U-net architecture from [30]. We use the residual error estimator from the first part of the paper as a criterion for the surrogate (encoder–decoder) model update. For further details see Section 3.5.

Let us summarize the three classes of exemplar problems studied in this paper. First, we study the eigenvalue problem for approximating the unique positive normalized ground state $u_{0}$. We aim to construct certified, robust and scalable—with respect to the increase in the dimension of the problem—approximation methods. Second, to approximate the eigenvalues higher in the spectrum we study the landscape function. The landscape function is obtained as a solution to the source problem Hu=1. It is again a positive smooth function and positivity is the only physical constraint needed to study the localization phenomena for the associated eigenstates. Further, we use simple residual control to ensure that the computed solution is a small perturbation of the true landscape function. As the third class of problems, we present the data-based surrogate model of the map connecting a class of potentials to the associated landscape functions. Here we are concerned with the use of convolutional networks as a data-sparse reduced order model in the context of certified surrogate modeling of this mapping. In particular, we are interested in the possibility of updating the surrogate model based on the residual error estimator.

## 2. Theoretical Background

In order to be precise and explicit, we will present the theoretical foundations on a somewhat restricted set of neural network architectures. The network architectures that will be used in practical computations are presented in Appendix B. The change of the family of the realizations of neural networks over which the optimization is carried out does not change the presentation of the algorithms in any practical way. Our main contribution is in the introduction of the error control in the Deep Ritz algorithm from [20]. We will now summarize the basic definitions from [31], which are necessary to interpret the numerical experiments.

**Definition** **1.** *Given $n_{1},n_{2},L\in N$, a neural network θ of depth D with the input dimension $n_{1}$ and the output dimension $n_{2}$ is the D-tuple $\theta =\left((A_{l},b_{l})\;:\;l=1,\cdots ,D\right)$ where:*

$$\left(A_{l},b_{l}\right)\in R^{N_{l}\times N_{l-1}}\times R^{N_{l}},\;\;l=1,\cdots ,D.$$

*By the convention $n_{1}=N_{0}$ and $n_{2}=N_{D}$. In the case in which D=2 we call the network shallow, and otherwise the network is called deep. The vector $\vec{N}=\left(\begin{array}{ccc} N_{0} & \cdots & N_{D} \end{array}\right)$ is called the network architecture of the neural network θ.*

We will use D(θ) to denote the depth of the given neural network θ. In the case in which the structure of matrices $A_{l}$, l=1,⋯,D is not further restricted, we say that the network is dense. In the case in which a sparsity pattern is assumed we have several subclasses of neural networks. For exemple, if the matrices $A_{l}$, l=1,⋯,D have a structure of a Toeplitz matrix ${\left(A_{l}\right)}_{ij}={\left(w_{i-j}^{l}\right)}_{ij}$—here $w^{l}$, l=1,⋯,D are parameter vectors defining a Toeplitz matrix [32]—we talk about convolutional neural networks.

Let ρ:R→R be a function that is not a polynomial. By ρ we denote the function $\rho ={⊗}_{l=1}^{n}\rho :R^{n}\to R^{n}$. We will now define a realization of the neural network θ with respect to the function ρ.

**Definition** **2.** *A function $R_{\theta ,\rho }:R^{n}\to R^{m}$ is defined by the formula:*

$$R_{\theta ,\rho }\left(x\right)=T_{L}\left(\rho \left(T_{D-1}\left(\rho \left(T_{D-2}\left(\cdots \rho \left(T_{1}\left(x\right)\right)\right)\right)\right)\right)\right)\;.$$

*where $T_{l}\left(x\right)=A_{l}x+b_{l}$, l=1,⋯,D is called the realization of the neural network θ with respect to the activation function ρ.*

Among various activation functions we single out the rectified linear unit (ReLU) function $\rho_{LU}=\max\left\{0,x\right\}$ and the sigmoid function $\rho_{S}\left(x\right)=1/\left(1-\exp\left(-x\right)\right)$. The set of all ReLU realizations of a neural network θ has a special structure. We call a function $f:R^{n_{1}}\to R^{n_{2}}$ piece-wise linear if there is a finite set of pairwise disjoint, closed polyhedra whose union is $R^{n_{1}}$ such that a restriction of *f* onto a chosen polyhedron is an affine function. It has been shown in [33] that any piece-wise linear function can be represented by a ReLU neural network and that any ReLU realization of a neural network is piece-wise linear. This observation is key to linking the approximation theory for neural networks with the standard Sobolev space regularity theory for partial differential equations.

Let us now fix some further notation. Let *m* be the number of the degrees of freedom of the space of piece-wise linear functions associated to the fixed polyhedral tessellation of Ω. We use $V_{m}$ to denote the set of all piece-wise linear functions on this tessellation. We also use the notations $P_{1}$, $P_{2}$ and $P_{3}$ to denote the space of piece-wise linear, quadratic and cubic functions, respectively. The corresponding interpolation operators (for continuous arguments) are denoted respectively by $I_{P_{1}}$, $I_{P_{2}}$ and $I_{P_{3}}$.

We will now briefly review a-posteriori error estimates that are used in this work. Let us note the following convention. We use $\varepsilon_{0}$ and $u_{0}$ to denote the ground state energy and the normalized positive ground state. We use $\varepsilon_{1}$ to denote the energy of a first excited state and we note that the notation is generalized for higher excited states in an obvious way. We denote the Rayleigh quotient of the operator *H* for the state ψ by ε=ε(ψ):=(ψ,Hψ)/(ψ,ψ). The standard Kato–Temple estimate from [26] can be written in a dimension-free form, also known as the relative form:

(4) $$\frac{|\varepsilon -\varepsilon_{0}|}{\left|\varepsilon \right|}\leq \frac{\left|\varepsilon \right|}{|\varepsilon_{1}-\varepsilon |}\frac{{∥H\psi -\varepsilon \psi ∥}^{2}}{{\left|\varepsilon \right|}^{2}{∥\psi ∥}^{2}}.$$

The quantity $\left|\varepsilon \right|/|\varepsilon_{1}-\varepsilon |$ is a measure of the so-called relative spectral gap [34,35] and it measures the distance to the unwanted part of the spectrum. It can be estimated by symmetry considerations or other a-priori information. In fact, a more careful analysis from [35] shows that the scaled residual is an asymptotically exact estimate of the relative error and so we will heuristically drop the measure of the gap even in the preasymptotic regime. A consequence of the Davis–Kahan theorem [36] is that the residual also estimates the eigenvector error:

(5) $$\frac{∥\psi -u_{0}∥}{∥\psi ∥}\leq c\frac{\left|\varepsilon \right|}{|\varepsilon_{1}-\varepsilon |}\frac{∥H\psi -\varepsilon \psi ∥}{|\varepsilon |∥\psi ∥}\;.$$

Subsequently, the error estimator $\Delta_{\varepsilon }^{2}={∥H\psi -\varepsilon \psi ∥}^{2}/(\varepsilon^{2}{∥\psi ∥}^{2})$ is a good stopping criterion for a certified approximation of an eigenmode.

For the source problem Hu=1, $u\in H_{0}^{1}\left(\Omega \right)$ we note the following relationship:

$$\frac{∥u-\underline{u}{∥}_{L^{2}}}{∥\underline{u}{∥}_{L^{2}}}\leq \frac{{∥1∥}_{L^{2}}}{\varepsilon_{0}}\frac{∥H\underline{u}{-1∥}_{L^{2}}}{∥\underline{u}{∥}_{L^{2}}{∥1∥}_{L^{2}}}\;,$$

between the residual and the relative $L^{2}$ error. Subsequently we use $\tau =∥H\underline{u}{-1∥}_{L^{2}}$/ $\left(∥\underline{u}{∥}_{L^{2}}∥1{∥}_{L^{2}}\right)$ as an error estimator for the source problem.

### Algorithms

We will now present the modifications of algorithms that we used to study the localization phenomena. We modified the Deep Ritz algorithm from [20] with the introduction of the a-posteriori (residual) error estimator. We call our variant the Certified Deep Ritz Algorithm. It is motivated by the work on certified reduced-order modeling in [29]. In order to be able to formulate strong residuals, we chose smooth activation functions ρ, so that $R_{\theta ,\rho }$ can be used to form the strong residual.

The performance of the stochastic gradient descent, when applied to the loss function, can be highly sensitive to the choice of the learning rate. Furthermore, it can also suffer from oscillation effects introduced by the choice of the sampling method in the numerical integration routines. For this reason we have opted to use the Adam optimizer from [37], which determines the learning rate by adaptively using information from higher-order moments.

To enforce the positivity constraint, we composed a realization of the neural network with the function Ξ, Ξ>0. We call the function Ξ a positivity mask and it must be chosen appropriately for the governing boundary conditions. As the positivity mask for Schrödinger Hamiltonians we either chose a smooth nonnegative function Ξ, which decays to zero as |x|→∞, or set the positivity mask as the identity.

In Algorithm 1 the parameter β>0 is the penalty parameter used to enforce the boundary conditions and the parameter η>0 is used to normalize the eigenmode approximation. We solve the integral using a Gauss quadrature rule in 1D and for higher dimensional problems we use quasi-Monte Carlo integration from [38,39] or a sparse grid quadrature [40] to approximate the integrals in the loss function as well as for the final (more accurate) evaluation of the energy functional. Alternatively in 2D, we sometimes choose to compute the integrals by projecting the realization of a neural network into a finite element space and then use the finite element quadrature to compute the integral. According to the authors of [11,41], this is an appropriate approach for problems of moderate dimensions ($\Omega \subset R^{n}$, n≤20). For higher-dimensional problems, Monte Carlo integration is the only scalable approach recommended.
**Algorithm 1:** Certified Deep Ritz Algorithm.
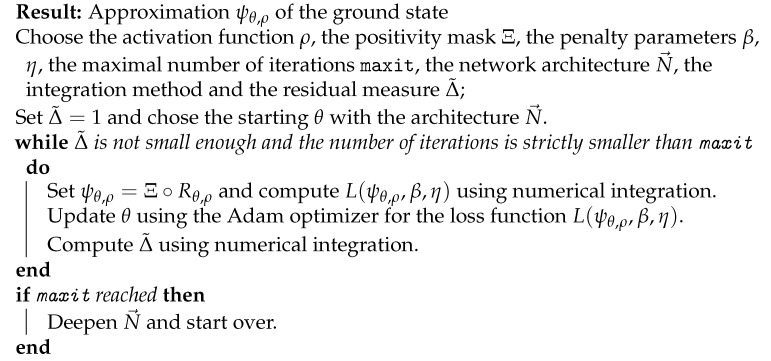


To apply Algorithm 1 to an eigenvalue problem we choose:

$$\tilde{\Delta }:=\Delta_{\varepsilon }^{2}=∥H\psi_{\theta ,\rho }-\varepsilon \left(\psi_{\theta ,\rho }\right)\psi_{\theta ,\rho }{∥}^{2}/(\varepsilon {\left(\psi_{\theta ,\rho }\right)}^{2}∥\psi_{\theta ,\rho }{∥}^{2})$$

and set the normalization parameter η>0 and β>0 for the loss function:

(6) $$L\left(u;\beta ,\eta \right)=\frac{\int_{\Omega }{\left|\nabla u\right|}^{2}+\int_{\Omega }V_{\omega }u^{2}}{\int_{\Omega }u}+\beta {∥u∥}_{L^{2}\left(\partial \Omega \right)}^{2}+\eta \left(\int_{\Omega }u^{2}-1\right)\;.$$

In the case of the source problem for the computation of the landscape function we set the normalization parameter η=0 and β>0 and define the loss function as:

$$L\left(u;\beta ,\eta \right)=\frac{1}{2}\left(\int_{\Omega }{\left|\nabla u\right|}^{2}+\int_{\Omega }V_{\omega }u^{2}\right)-\int_{\Omega }u+\beta {∥u∥}_{L^{2}\left(\partial \Omega \right)}^{2}\;.$$

As the error indicator we take $\tilde{\Delta }=\tau =∥H\underline{u}{-1∥}_{L^{2}}/(∥\underline{u}{∥}_{L^{2}}{∥1∥}_{L^{2}})$, where $\underline{u}=\Xi \circ R_{\theta ,\rho }$. Here we have chosen as an example the homogeneous Dirichlet boundary condition $u{|}_{\partial \Omega }=0$. Other self-adjoint boundary conditions can equally be implemented by penalizing the boundary conditions residual at the boundary ∂Ω. Note that computing derivatives of realizations of neural networks is efficiently implemented in many programming environments such as TensorFlow [42].

## 3. Results

In this section we present direct approximation methods for estimating the ground state $u_{0}$, the ground state energy $\varepsilon_{0}$ and the landscape function *u*. We use the Certified Deep Ritz Algorithm presented as Algorithm 1.

### 3.1. Direct Approximations of the Ground State in 1D

We now present the results of the application of Algorithm 1 on the 1D Schrödinger operator $H=-\partial_{xx}+V.$ For the domain Ω=(−B,B), we choose the loss function (6) and set $\Xi \left(x\right)=\exp\left(-x^{2}/10\right)$ as the positivity mask in Algorithm 1.

To benchmark the accuracy of the VPINN approximations we have solved the problem to high relative accuracy using the Chebyshev spectral method as implemented in the package chebfun [43,44]. We emphasize that chebfun was not used during the training of the network in any way. To compute the residuals and the energy of the ground state we used a Gaussian quadrature where the deep network is evaluated at the sufficient number—for the given interval (−B,B)—of Gaussian points.

We constructed the potential *V* as a linear combination of the finite well and two inverted Gaussian bell functions:

$$V=-\alpha_{1}\exp(-∥\cdot -c_{1}{∥)}^{2}/k_{1}^{2})-\alpha_{2}\exp(-∥\cdot -c_{2}{∥)}^{2}/k_{2}^{2})-h1_{\left\{x:\;|x-c|<t\right\}}.$$

**Figure 1:**
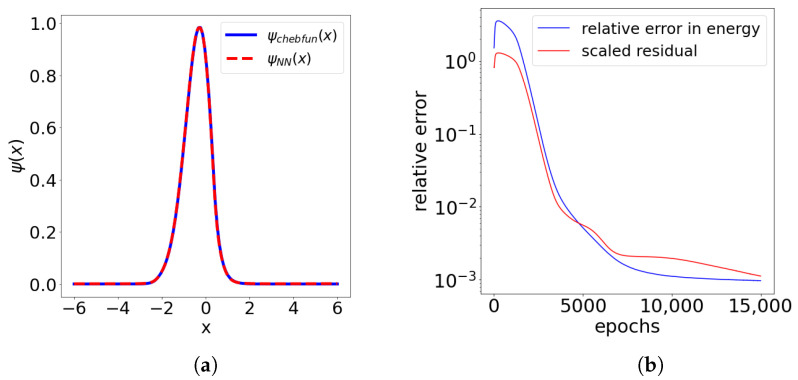
(**a**) Comparison of the ground state obtained in chebfun ($\psi_{chebfun}\left(x\right)$) and as the VPINN solution ($\psi_{NN}\left(x\right)$) with the architecture ${\vec{N}}_{\mathrm{DenseNet}}=\left(n,k,l,m\right)=\left(1,4,2,10\right)$; (**b**) Residual and Rayleigh quotient error estimate metrics during the training process.

**Figure A1:**
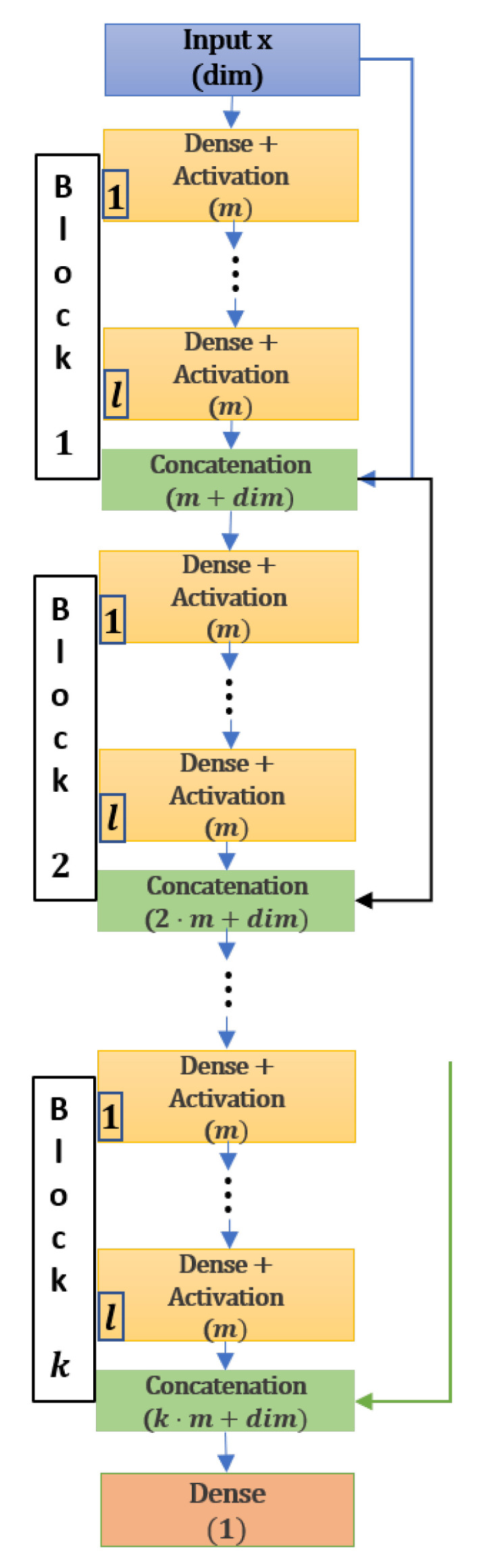
VPINN architecture with *k* blocks, *l* layers in each block and *m* neurons in each dense layer.

We used $1_{\left\{x:\;|x-c|<t\right\}}$ to denote the indicator function and chose $\alpha_{i}\in \left[8,12\right]$, $c_{i}\in \left[-2.5,2.5\right]$, $k_{i}\in \left[0.9,2.6\right]$, i=1,2, h∈[10,15], c∈[−2,2], t∈[0.5,2.5]. The neural network has 1162 trainable parameters and we used the DenseNet VPINN architecture ${\vec{N}}_{\mathrm{DenseNet}}\left(1,4,2,10\right)$ with the activation function $\rho \left(x\right)=\exp\left(-x^{2}/10\right)$; see Figure A1. Figure 1 shows the solution and the error estimate during 15,000 epochs of a run of the Adam optimizer with the learning rate $\delta =10^{-3}$ and a batch size of 1024. In this example we used 1024 quadrature points on an interval (−6,6) and the penalty parameters $\beta =10^{-3}$ and η=20.

One can observe robust, almost asymptotically exact, performance of the estimator:

$$\Delta_{\varepsilon }^{2}={∥H\psi -\varepsilon \psi ∥}^{2}/(\varepsilon^{2}{∥\psi ∥}^{2})\;.$$

Let us also emphasize that $\Delta_{\varepsilon }$ measures the distance of the Rayleigh quotient (energy functional) to the nearest eigenvalue. For evaluation of the integrals in higher dimensions we refer the reader to Appendix B. Note that the final error for the approximation of the ground state energy was 0.1%, whereas the final relative $L^{2}$ error in the ground state was 0.9%. This is in line with the eigenmode error estimate (5).

### 3.2. Direct Approximations of the Ground State in Higher-Dimensional Spaces

We present VPINN approximation results for the ground state and the ground state energy of the Schrödinger equation with harmonic oscillator potential $V\left(x\right)={∥x∥}^{2}$ using the dense network with the architecture depicted in Figure A1. We study the problem on the truncated domain ${\left[-3,3\right]}^{n}$, where *n* is the dimension of the space. Neural network architecture should be constructed with caution. There are multiple sources of instability when dealing with neural networks, e.g., exploding and vanishing gradients. We experimented with a variety of different activation functions: $\rho_{S}\left(x\right)$, $\rho_{LU}{\left(x/10\right)}^{2}$, $x\rho_{S}\left(x\right)$, $\exp(-x^{2}/10)$ etc. After training of the neural network using the quasi-Monte Carlo realization of the energy integrals to define the loss function we computed the approximate ground state energy using the approximation of the energy functional (Rayleigh quotient) using the Sobol sequences with 100,000 points. We also report on the results obtained using Smolyak grids of order 6 with the Gauss–Patterson rule. The results are presented in Table 1.

The accuracy of the ground state energy approximations that were obtained using quasi-Monte Carlo integration are comparable with the accuracy reported in [20]. On the other hand, the results obtained by using Smolyak’s points were unsatisfactory in dimensions higher than 3. This observation will be the subject of future research. It appears that the oscillation of the realizations of the neural network on the boundary of the computational domain together with the appearance of negative weights in sparse grid integral formulas contributed to the instability of the approach. We used the $\vec{N}=\left(n,5,2,20\right)$ architectures for n≤6 and $\vec{N}=\left(9,3,1,10\right)$ in 9D and the swish function $\rho \left(x\right)=x\rho_{S}\left(x\right)$ as the activation function. The positivity mask was chosen as the identity.

### 3.3. Approximations of the Landscape Function in 1D

**Figure 2:**
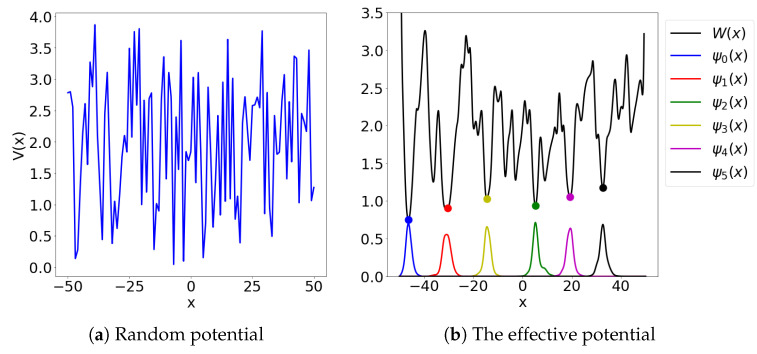
The effective potential and its 6 local minima, which define localization of the first six eigenstates is shown on the right. Eigenstates $\psi_{i},i=0,1,...,5$ were computed in chebfun.

We now present the result of the approximation method using the landscape function as a solution of the partial differential equation Hu=1, $u\in H_{0}^{1}\left(\Omega \right)$. The potential $V\in C\left(\left[-50,50\right]\right)$ is constructed as a random piece-wise linear function (see Figure 2). More to the point, let $\xi_{k}$, k=−50,⋯,50 be independently drawn numbers from the uniform distribution on the interval $\left[0,4\right]$. We construct the potential *V* as the piece-wise linear interpolant of $\left(k,\xi_{k}\right)$, k=−50,⋯,50. We again used chebfun for benchmarking. We set η=0 and defined the loss function as:

(7) $$L\left(u;\beta ,\eta \right)=\frac{1}{2}\left(\int_{\Omega }{\left|\nabla u\right|}^{2}+\int_{\Omega }V_{\omega }u^{2}\right)-\int_{\Omega }u+\beta \left(u{\left(-50\right)}^{2}+u{\left(50\right)}^{2}\right)\;.$$

Here we have used the architecture of the dense VPINN network; see Appendix C. We used β=500 for the experiments. In Figure 2 we can see the six local minima of the effective potential W=1/u obtained from the neural network and the first six eigenstates computed by the chebfun. Note that the potential W=1/u is defined only in the interior of the domain (−50,50). In Table 2 we present the results of the benchmarking of the approximation formula (2) against highly accurate chebfun eigenvalue approximations.

The neural network has 6402 trainable parameters and we used the VPINN architecture ${\vec{N}}_{\mathrm{DenseNet}}=\left(1,5,2,20\right)$ with the activation function $\rho_{LU}{\left(x/10\right)}^{2}$. The positvitiy mask was chosen as the identity. The network was trained using 50,000 epochs of the Adam optimizer with the learning rate $\delta =10^{-3}$ and a batch size of 2048. In this example we also used 2048 quadrature points on an interval (−50,50).

### 3.4. Direct VPINN Approximation of the Landscape Function in 2D

We now apply Algorithm 1 to the problem of approximating the landscape function in 2D. When presenting the examples we will report on the used network architecture ${\vec{N}}_{\mathrm{DenseNet}}=\left(2,k,l,m\right)$ as well as indicate the number of trainable parameters for each of the architectures. The activation function used for all neural networks in this subsection is the sigmoid function $\rho_{S}$. We now set l=2 and in the next table present a convergence study for the family of architectures ${\vec{N}}_{\mathrm{DenseNet}}=\left(2,k,2,m\right)$.

The convergence histories of relative $L^{2}$ and $H^{1}$ errors, measured with respect to the benchmark FEniCS solution, are shown in Table 3. We can observe that the errors drop at a favorable rate with an increase in *k*. On the other hand, an increase in *m* causes a much more pronounced increase in the number of trainable parameters (complexity of the network) but incurs, in comparison, only a moderate improvement of the accuracy level.

**Figure 3:**
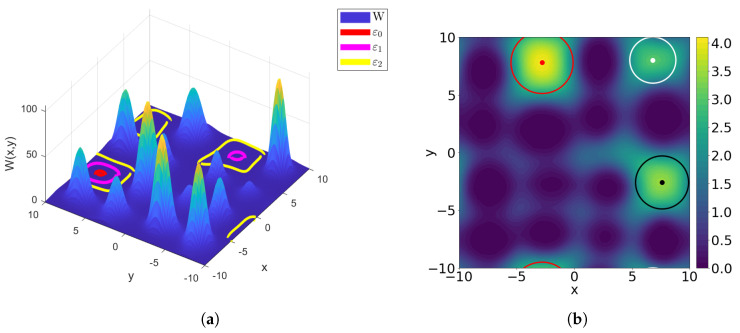
A surface plot of the effective potential W=1/u (**a**) and the landscape function *u* (**b**). In (**a**) we plot the boundaries of the sets {x:εu(x)≥1} that localize the eigenstates. In (**b**) we plot the circles of radius $1/\tilde{\varepsilon_{i}}$, for ${\tilde{\varepsilon }}_{i-1}=3W_{\min,i}/2$, i=1,2,3, centered at the *i*-th lowermost local minimum $W_{\min,i}$.

In Figure 3 we plot the effective potential *W* and the landscape function *u*.

### 3.5. Encoder–Decoder Network as a Reduced-Order Model for a Family of Landscape Functions

We now study the use of the sparse, U-Net-inspired [30], network architecture as a surrogate model for the function L:V↦u. In Figure 3 we show the landscape function *u* with periodic boundary conditions for the potential $V_{\omega }$ constructed as a lattice superposition of sixteen Gaussian bell functions $G\left(x\right)=\alpha \exp(-|x_{1}-c_{1}{|}^{2}/k_{1}^{2}-|x_{2}-c_{2}{|}^{2}/k_{2}^{2})$. The centers $c=(c_{1},c_{2})$ were chosen randomly inside each block of the 4×4 uniform quadrilateral tessellation of Ω. The constants $\alpha ,k_{i},i=1,2$ were chosen randomly and independently from intervals [8,128] and [1,1.5], respectively. To introduce local defects in the lattice, we have further randomly chosen three Gaussian bells and removed them from the potential. The choice of Gaussian bells to be removed was restricted, so that the boundary conditions were respected and that none of the erased bells were pairwise adjacent.

**Figure A2:**
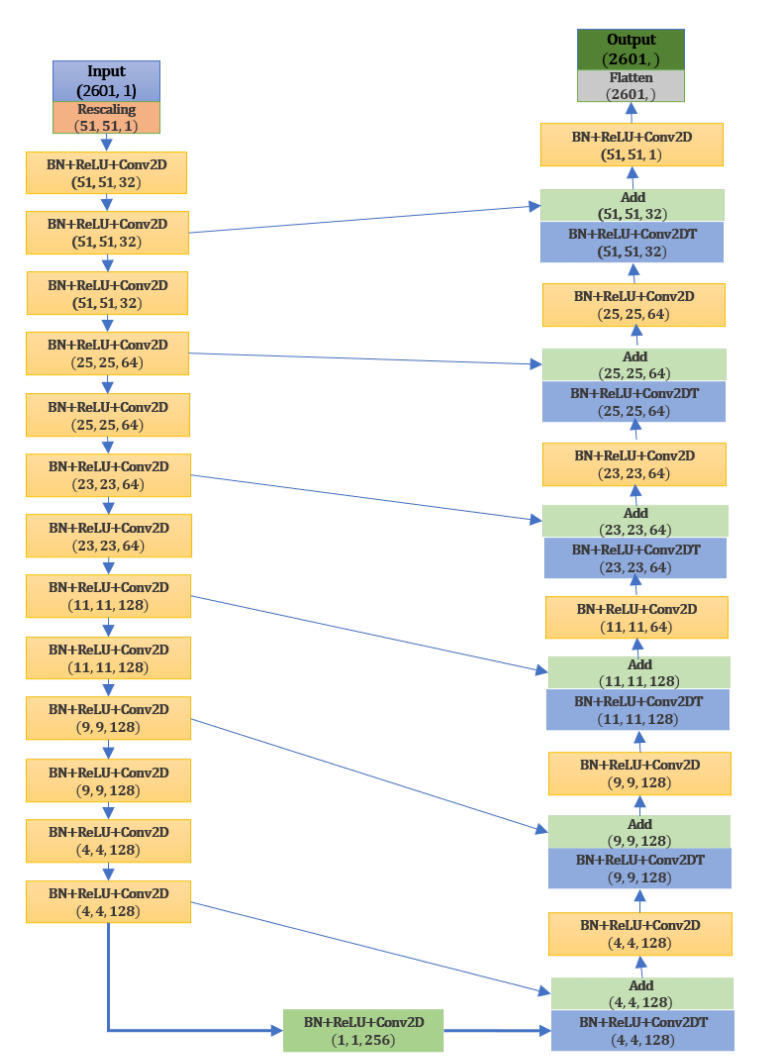
FCNN encoder–decoder architecture inspired by the U-Net concept from [30].

As the reduced order model for this family of problems, we have used the encoder–decoder fully convolutional neural network (FCNN) from Appendix C with 2,614,531 trainable parameters. This is a relatively small number of parameters in comparison with the typical convolutional neural network architectures with fully connected layers. The architecture of the neural network is shown in Figure A2.

To train the model we generated 98,400 potentials $V_{\omega }$ and then used FEniCS to compute the associated landscape functions $u_{\omega }$, $-\Delta u_{\omega }+V_{\omega }u_{\omega }=1$. The domain of the Hamiltonaian was $\Omega ={\left[-10,\;10\right]}^{2}$, with the periodic boundary conditions. We used the uniform quadrilateral discretization with the step size h=20/50=0.4 and $P_{2}$ elements to compute the training examples. To construct the reduced-order model, we projected (by interpolation) these $P_{2}$ functions onto the space of $P_{1}$ elements for the same mesh. After implementing the periodic boundary conditions we obtained exactly 2500 free nodes for this space of $P_{1}$ functions.

We denote the values of the potential *V* in those nodes as the vector $\vec{V}\in R^{2,500}$ and we tacitly identify the vector $\vec{V}$ with the function $I_{P_{1}}V$. Let $I_{P_{1}}^{*}$ be the extension operator from $R^{2500}$ to the space of continuous piece-wise linear functions. Then

$$\tilde{L}\left(\vec{V}\right):=I_{P_{1}}L\left(I_{P_{1}}^{*}I_{P_{1}}V\right)$$

defines the mapping $\tilde{L}:R^{2,500}\to R^{2,500}$. We used the learning rate $\delta =10^{-4}$ for the first 100 epochs of the Adam optimizer and the learning rate $\delta =10^{-5}$ for a further 50 epochs. The activation function $\rho_{LU}$ was used to promote sparsity and the MSE loss function was used for the training. The batch size for the Adam algorithm was 1024. We implemented the certified surrogate modeling approach by combining the evaluation of the neural network with the error estimator τ. In the case in which the residual measure τ for the function $\underline{u}=L\left(I_{P_{1}}^{*}I_{P_{1}}\hat{V}\right)$ is larger than the preset tolerance, we updated the surrogate model (neural network). To this end we solved in FEniCS the problem $-\Delta u+\hat{V}u=1$ and used the standard update algorithm for the convolutional neural network and the new training example.

**Figure 4:**
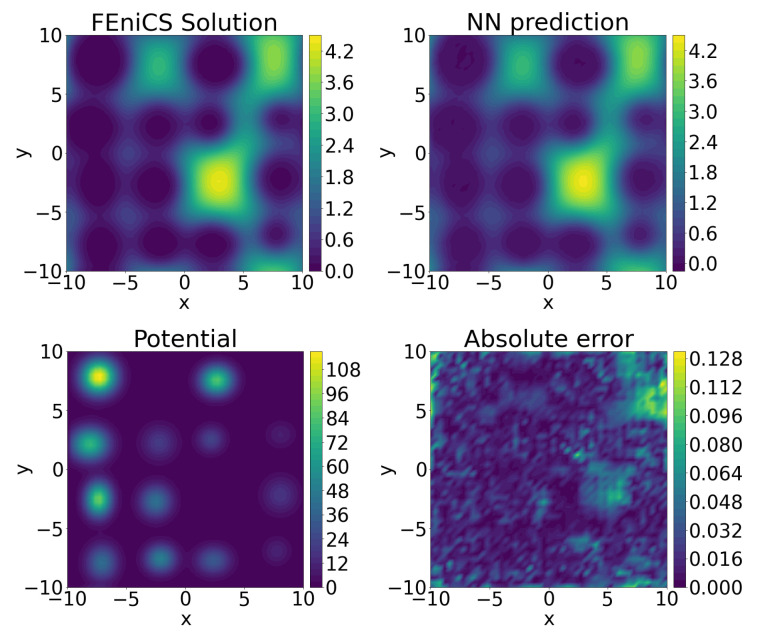
A benchmarking comparison of the encoder–decoder prediction of the landscape function against the FEniCS solution.

We evaluated the performance of the neural network reduced-order model on a set of 200 testing potentials that were not used in the training of the network, see Table 4. The benchmarking comparison against the FEniCS solution is presented in Figure 4.

## 4. Discussion

According to the authors of [45], deep learning approaches to dealing with partial differential equations fall into the following three categories: (a) Rayleigh–Ritz approximations, (b) Galerkin approximations and (c) least squares approximations. We have considered a hybrid approach that combines robust stochastic optimization of overparametrized networks based on the Rayleigh–Ritz approach with standard residual based error estimates, which together yield a hybrid approximation method. We were particularly influenced by the review in [45] and the Deep Ritz algorithm as described in [20].

**Figure 5:**
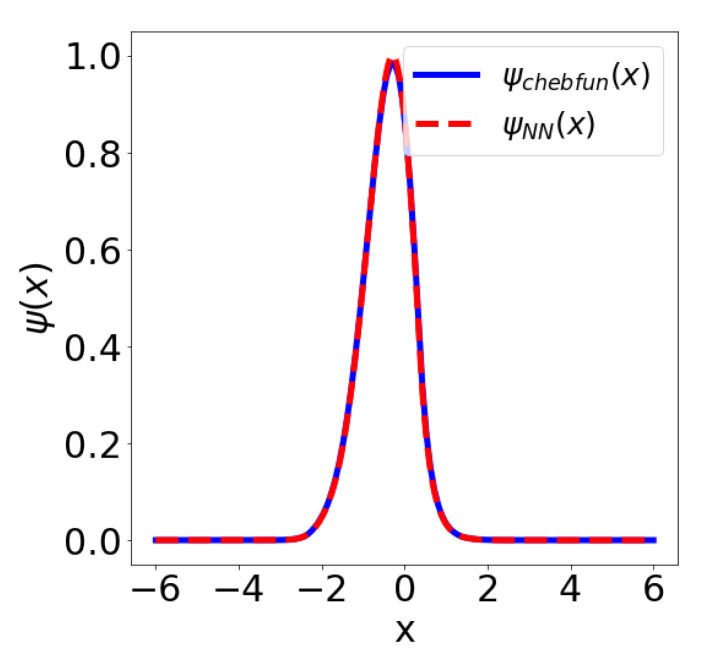
Comparing the Chebyshev series expansion with 149 terms and a VPINN solution with the architecture ${\vec{N}}_{\mathrm{DenseNet}}=\left(1,\;2,\;2,\;2\right)$ and 30 trainable parameters.

In the example from Table 3 we further computed a piece-wise cubic and piece-wise quadratic approximation of the landscape function using the standard finite element method and an approximation of the landscape function using a variant of the Deep Ritz method. We measured the error of the $P_{2}$ and the VPINN approximation against the $P_{3}$ benchmark solution. The relative $L^{2}$ error of the piece-wise quadratic approximation was computed to be 0.36%, whereas the relative error of the VPINN approximation with 1203 free parameters was computed to be 1.21%. Note, however, that the piece-wise quadratic approximation required the training of 10,000 parameters. This indicates that the neural network achieves a considerable data compression when compared with a piece-wise polynomial approximation. The situation is even more interesting in 1D. There we compared the ground state approximation by the Chebyshev series, as implemented in chebfun. We needed 149 terms in the Chebyshev expansion to reach the order of machine precision. On the other hand, the Adam optimizer was able to find a realization of the dense neural network with VPINN architecture ${\vec{N}}_{\mathrm{DenseNet}}=\left(1,\;2,\;2,\;2\right)$ from Appendix A. This architecture only has 30 trainable parameters and it achieves a relative distance (in the $L^{2}$ sense) of 1.05% to the benchmark chebfun solution. The relative error in the ground state energy is only 0.1%, see Figure 5.

The integrals needed to approximate the energy functional were computed using Gaussian quadrature rules. Unfortunately, this approach does not yield stable methods in higher-dimensional problems. The reason is in the fact that sparse grid quadratures (e.g., [40]) also have nodes with negative weights and this was observed to cause severe numerical instability. An approach based on the quasi-Monte Carlo integration, which utilizes low-discrepancy sequences of integration nodes and has only positive weights (see [39]) yielded an efficient and stable method in higher-dimensional situations. Furthermore, since realizations of neural networks are frequently functions with many local extrema, computing their integrals needs to be handled with care. This is particularly relevant when enforcing discretizations of physical or normalization constraints by penalization. We point out that the scalability of sparse-grid integration schemes in this respect was not satisfactory.

Another promising technique for obtaining data-sparse compressed approximation of the solutions of partial differential equations is based on the concept of tensor networks, also known as matrix product states or tensor train decompositions [9]. This approach has been successfully converted into numerical approximation algorithms such as the quantized tensor train decompositions [8,46]. The scaling robust performance of this approach has been demonstrated on a class of multi-scale model problems in [46]. However, the numerical methods still have to be tailor-made for the chosen problems. On the other hand, there are many freely available robust and highly developed libraries for working with deep neural networks. This is the reason for our choice of the discretization method.

## 5. Conclusions

We have presented two types of neural network architectures. First, a dense deep network of the DenseNet type was used as a compressed approximation of the ground state and the landscape function of the problems under consideration. Remarkably, it achieved high accuracy and a good compression rate even when empirically compared with a Chebyshev expansion in 1D. Even though we managed to tackle problems in $R^{n}$, this concept struggled to yield scalable numerical methods. We then took another approach and considered a problem of approximating a mapping $L:R^{2500}\to R^{2500}$, which connects a mesh sample of a potential with the associated landscape function. A fully convolutional neural network architecture with the ReLU activation function, to further promote sparsity, turned out to be expressive enough to deal with this family of problems to a satisfactory level of accuracy (empirically measured on the test set). We have also seen that a hybrid approach—one that combines the expressivity of the set of neural network realizations with the standard error indicators—has a potential to lead to robust approximation methods. We have observed that it is particularly challenging to turn physical constraints—which are continuous—into their discrete realizations, which can be used to filter out, e.g., by judiciously applied penalization, the nonphysical neural network realizations from the set of all realizations of a given architecture. How to turn this into a robust mesh-less and scalable method for dealing with equations of mathematical physics will be a topic of further research.

## Figures and Tables

**Table 1:** Convergence rates for the ground state energy of the harmonic oscillator in relation to the dimension. QMC: quasi-Monte Carlo.

*n*	$\varepsilon_{0}$	*M* for the Loss Function	Adam Optimizer Epochs	*M* for the Smolyak Quadrature	Smolyak Relative Error %	Relative Error for QMC with $M=10^{5}$ Points%
1	1	100	50,000	127	0.004	0.003
2	2	1000	20,000	769	1.416	1.226
3	3	5000	50,000	2815	1.110	1.608
6	6	50,000	80,000	40,193	-	1.40
9	9	50,000	50,000	242,815	230.366	5.816

**Table 2:** We tested the accuracy of the predictor ${\tilde{\epsilon }}_{i-1}=\left(1+\frac{n}{4}\right)W_{min,i}$ for 16 lowermost eigenvalues. The chebfun solution was used to benchmark the error.

	**0**	**1**	**2**	**3**	**4**	**5**	**6**	**7**
Minimum values of *W*	0.747201	0.918677	0.918754	0.933014	1.028903	1.057663	1.174706	1.245278
chebfun eigenvalues	0.979730	1.071839	1.230230	1.282611	1.301724	1.485232	1.577349	1.588252
Relative error in %	4.6675	7.1379	6.6481	9.0708	1.1981	1.9850	6.9082	1.9930
	**8**	**9**	**10**	**11**	**12**	**13**	**14**	**15**
Minimum values of *W*	1.256498	1.273980	1.326926	1.613203	1.848415	1.868003	1.907063	1.931723
chebfun eigenvalues	1.625253	1.758768	1.780166	2.095899	2.161778	2.265704	2.270798	2.278380
Relative error in %	3.3614	9.4551	6.8257	3.7882	6.8805	3.05864	4.9776	5.9811

**Table 3:** A report on the convergence in *k* and *m* for the family of architectures ${\vec{N}}_{\mathrm{DenseNet}}=\left(2,k,2,m\right)$. We benchmark the error against the highly accurate $P_{3}$ FEniCS solution.

Parameters	*k*	*m*	Relative $L^{2}$ Error 100,000 Epoch	Relative $H^{1}$ Error 100,000 Epoch	Relative $L^{2}$ Error 200,000 Epoch	Relative $H^{1}$ Error 200,000 Epoch	Relative Error of the First Three Eigenvalues Respectively
803	4	8	2.5852%	5.6216%	2.0527%	4.9876%	0.1638%, 1.4479%, 1.1472%
1203	4	10	2.7487%	5.3611%	1.2354%	3.6960%	0.0839%, 2.3489%, 0.6341%
1753	5	10	1.9314%	4.2386%	1.0679%	3.3851%	0.5957%, 1.9264%, 0.3822%
2403	6	10	1.1745%	3.0548%	0.7998%	2.6994%	0.4539%, 1.7883%, 1.5112%
4403	4	20	1.9037%	3.6929%	0.7233%	2.5757%	0.3242%, 1.8831%, 1.2586%
9603	4	30	1.8217%	3.7451%	0.6689%	2.3609%	0.3639%, 2.0083%, 0.9685%
16,803	4	40	0.6372%	1.9704%	0.3920%	1.5497%	0.3269%, 1.8606%, 0.6983%
26,003	4	50	3.6993%	7.3510%	0.4207%	1.6748%	0.3127%, 1.5756%, 0.3559%

**Table 4:** Validation of the encoder–decoder representation of the mapping L:V↦u on a collection of test examples. Recall that the effective potential is defined as W=1/u.

Average $L^{2}$ error	1.7545%
Maximal $L^{2}$ error	2.9769%, example: 58
Average $H^{1}$ error	9.2233%
Maximal $H^{1}$ error	12.6765%, example: 65
Mean relative error in $1/W_{\min,1}$	0.4887%
Maximal relative error in $1/W_{\min,1}$	2.1402%, example: 70
The worst ten relative errors in $1/W_{\min,1}$ (%)	2.1402, 1.5909, 1.5560, 1.4816, 1.4151, 1.4626, 1.3441, 1.3377, 1.3181, 1.3132

## Sample Availability

Codes are available from the GitHub repository https://github.com/markohajba/NN-Schrodinger. All communication regarding software should be directed to marko.hajba@vsmti.hr.

## Abbreviations

   The following abbreviations are used in this manuscript:

PDE	partial differential equation
ReLU	rectified linear unit
FEM	finite element method
DOF	degrees of freedom
VPINN	Variational Physics Informed Neural Networks
FCNN	fully convolutional neural network

## Appendix A. Implementation Details

In the implementation of the discussed methods we have used the following tools in the Python programming environment: TensorFlow 2 [42], Keras [47], chaospy [38] and FEniCS [48]. Implementations and additional materials are available at GitHub repository https://github.com/markohajba/NN-Schrodinger.

## Appendix B. Estimating Residuals

The Rayleigh quotient of the operator *H* for the mode ψ is computed by evaluating the integral:

$$\varepsilon \left(\psi \right)=\frac{\int_{\Omega }\nabla \psi \cdot \nabla \psi +V\psi^{2}}{\int_{\Omega }\psi^{2}}\;.$$

The eigenvalue residual for the mode ψ is the functional:

$$\phi \mapsto \left(H\psi -\varepsilon \left(\psi \right)\psi ,\phi \right)=\int_{\Omega }\left(\nabla \psi \cdot \nabla \phi +(V-\varepsilon (\psi ))\psi \phi \right)\;.$$

We measure the norm of the residual by approximating the supremum:

$$\underset{\phi \in S\subset H^{1}\left(\Omega \right)}{\sup}\frac{\left|(H\psi -\varepsilon (\psi )\psi ,\phi )\right|}{∥\phi ∥}$$

over a judiciously chosen set S. This residual can also be written as a functional and approximated by solving the optimization problem over S. We leave out the details.

In the original Deep Ritz Algorithm from [20] the authors have used Monte Carlo integration to compute the duality products. This amounts to choosing a random sample $\chi_{i}\in \Omega$, i=1,⋯,M and then using:

$$\int_{\Omega }{∥\nabla \psi ∥}^{2}+V\psi^{2}\approx \frac{1}{M}\sum_{i=1}^{M}\left(∥\nabla \psi \left(\chi_{i}\right){∥}^{2}+V\psi {\left(\chi_{i}\right)}^{2}\right)\;.$$

By contrast, in $R^{n}$, we used the Smolyak quadrature [38,40] and low-discrepancy sequences for quasi-Monte Carlo integration routines [39]. For a given order of the quadrature there exist weights $\alpha_{i}\in R$ and nodes $\xi_{i}\in \Omega$, i=1,⋯,M such that:

$$\int_{\Omega }{∥\nabla \psi ∥}^{2}+V\psi^{2}\approx \sum_{i=1}^{M}\alpha_{i}\left(∥\nabla \psi \left(\xi_{i}\right){∥}^{2}+V\psi {\left(\xi_{i}\right)}^{2}\right)\;.$$

Unlike in the Monte Carlo approach, these nodes are fixed (for a given choice of parameters, see [38]).

### Appendix B.1. Finite Element Quadrature for 2D Problems

For 2D problems we also used finite element quadrature to estimate the integrals and also to estimate the negative-order Sobolev norm. This approach does not scale to higher-dimensional problems, but is a good method for the purposes of validating algorithms based on the variational optimization and neural networks. Let $V_{h}\subset H_{\pi }^{1}\left(\Omega \right)$ be a finite element space and let $W_{h}$ be another finite element space such that $V_{h}\subset W_{h}\subset H_{\pi }^{1}\left(\Omega \right)$. To $V_{h}$ and $W_{h}$ we associate standard interpolation operators $I_{V_{h}}:C\left(\Omega \right)\to V_{h}$ and $I_{W_{h}}:C\left(\Omega \right)\to W_{h}$. For a given continuous realization of the neural network $\psi_{\theta }=\Xi \circ R_{\theta ,\rho }$, we compute:

$$\int_{\Omega }∥\nabla \psi_{\theta }{∥}^{2}+V\psi_{\theta }^{2}\approx \int_{\Omega }{∥\nabla \left(I_{V_{h}}\psi_{\theta }\right)∥}^{2}+V{\left(I_{V_{h}}\psi_{\theta }\right)}^{2}$$

and we use standard finite element quadratures to evaluate the integrals on the right-hand side, see for instance the FEniCS book [48].

Finally, to assess the negative-order Sobolev norm of the residual we use the auxiliary subspace $W_{h}$ and compute, using standard finite element calculus:

$$\sup_{\psi \in H_{\pi }^{1}\left(\Omega \right)}\frac{\left|\int_{\Omega }\left(\nabla \psi \cdot \nabla \phi +\left(V-\varepsilon \left(\psi \right)\right)\psi \phi \right)\right|}{∥\phi ∥}\approx \sup_{\psi \in W_{h}}\frac{\left|\int_{\Omega }\left(\nabla \left(I_{V_{h}}\psi \right)\cdot \nabla \phi +\left(V-\varepsilon \left(I_{V_{h}}\psi \right)\right)\left(I_{V_{h}}\psi \right)\phi \right)\right|}{∥\phi ∥}\;.$$

### Appendix B.2. Direct Approximations for Higher Dimensional Problems

For higher-dimensional problems we use quadrature rules based on low discrepancy sequences [39] to compute the value of the energy functional (Rayleigh quotient) on the returned neural network realization $R_{\theta ,\rho }$. To define the loss function, which consists of the energy functional and the normalization constraints, we use the quasi-Monte Carlo approach [49], but with fewer quadrature nodes. This is consistent with the approach taken in the original Deep Ritz algorithm. The use of Smolyak’s rules can also be considered. This, however, does not lead to numerically stable optimization procedures. The realizations of neural networks are functions that can have many sharp local extrema and so optimizing using a fixed collection of nodes was observed to lead to degenerate solutions. The further problem stems from the fact that Smolyak’s rules have, unlike Gaussian rules, a certain percentage of negative weights and so due to approximation errors it is possible to compute a negative approximation of an integral of a positive function. This is highly undesirable for a minimization procedure. Quasi-Monte Carlo integration routines are much less accurate than sparse grid rules, but all of their integration weights are positive and in addition they avoid the problem of overfitting. We use Sobol’s points [39] to calculate nodes for the quasi-Monte Carlo approximation of the energy functional for higher-dimensional problems.

## Appendix C. Architecture of the VPINN Neural Network

We will present the architectures of deep neural networks used in the paper. The network architecture $\vec{N}$ as defined in Definition 1 is sufficient to describe deep dense neural networks. Neural network architectures are presented graphically. Some further formal descriptions of the approximation classes can be found in [50]. The particular architecture that we use will have slightly more regularity in the dimensions of the layers. However, there will be more links between layers, which are inspired by the DenseNet concept [51]. The architecture is depicted in Figure A2 and we use the vector ${\vec{N}}_{\mathrm{DenseNet}}=\left(n,k,l,m\right)$ to describe the architecture of the network, which has *k* blocks with *l* layers of the size *m*. Realizations of this network are functions from $R^{n}$ to R.
